# Serum C-reactive protein to albumin ratio and mortality associated with peritoneal dialysis

**DOI:** 10.1080/0886022X.2020.1783680

**Published:** 2020-06-30

**Authors:** Siyi Liu, Panlin Qiu, Laimin Luo, Lei Jiang, Yanbing Chen, Caixia Yan, Xiaojiang Zhan

**Affiliations:** Department of Nephrology, The First Affiliated Hospital of Nanchang University, Nanchang, China

**Keywords:** Serum C-reactive protein to albumin ratio, peritoneal dialysis, mortality, serum C-reactive protein, albumin

## Abstract

**Background:**

Serum C-reactive protein to albumin ratio (CAR) was recently identified as a poor marker of prognosis among various populations. The current study aimed to examine the association between CAR and all-cause mortality among patients undergoing peritoneal dialysis (PD).

**Methods:**

A total of 758 patients with PD were included in this study during the period from 1 November 2005 to 28 February 2017 and followed up until 31 May 2017. The primary outcome was all-cause mortality. We used multivariate Cox proportional hazard models and Kaplan-Meier survival curves to assess the relationship between CAR and all-cause mortality in these patients.

**Results:**

Among 758 participants, mean age was 49.1 ± 14.2 years, with 56% males and 18.6% prevalence of diabetes. Median CAR was 0.13 (interquartile range [IQR], 0.07–0.34). After 27 months (IQR, 14–40 months) of follow-up, 157 deaths had been reported. After adjusting for confounding factors, we found a significant association between serum CAR and all-cause mortality among those in the highest CAR group (hazard ratio 1.91, 95% confidence interval 1.05– 3.47, *p* = 0.034).

**Conclusions:**

In patients undergoing PD, an increase in serum CAR is independently associated with increased risk for all-cause mortality.

## Introduction

Patients undergoing peritoneal dialysis (PD) have increased mortality risk because of increases in their risk for cardiovascular diseases (CVD), infections, and malnutrition. In addition to traditional CVD risk factors including hypertension, diabetes mellitus (DM), and dyslipidemia, which are prevalent among patients with PD, the presence of chronic inflammation may further increase the risk of mortality [1,2].

Serum C-reactive protein (CRP) and albumin (ALB) are both useful prognostic markers for assessing the mortality of patients with PD [3,4]. CRP levels reflect the severity of inflammation, while ALB may be used as a nutritional marker in critically ill patients [5]. Prior studies suggested an association between one’s nutritional status and the degree of inflammation; such an association may influence the risk of developing complications. Serum C-reactive protein to albumin ratio (CAR), a composite indicator of inflammation and nutritional status, has recently been recognized as an independent prognostic marker for use in patients receiving parenteral nutritional support due to critical illness, malignancy, infection, anti-neutrophil cytoplasmic antibody (ANCA)-associated vasculitis, or acute kidney injury [5–10]. However, few relevant studies have investigated use of this index in patients undergoing PD. Based on these results, we suspect that higher serum CAR values may predict increased mortality among patients with PD.

In this retrospective cohort study, we evaluated the relationship between serum CAR levels and all-cause mortality among patients with PD who were followed for an average of 27 months at our PD center.

## Materials and methods

### Study population and data collection

This study was conducted in accordance with the ethical principles of the Helsinki Declaration [11] and approved by the Human Ethics Committees of Nanchang University (application ID: [2019]088).

This study included patients receiving PD as their first modality of renal replacement therapy. Patients were followed up at the PD center of The First Affiliated Hospital, Nanchang University, Jiangxi, China during the period from 1 November 2005 through 28 February 2017. The inclusion criteria were as follows: age ≥ 18 years at the time of PD initiation and survival for ≥ 90 days since the day of PD initiation. Patients who were catheterized in other hospitals, transferred from permanent hemodialysis (HD), had failed renal allografts, or did not have baseline CRP or ALB data were excluded from the study. All patients were followed up until cessation of PD, death, or 31 May 2017, whichever occurred first. The baseline demographic data collected included age, sex, primary cause of end-stage renal disease (ESRD), and the presence of DM. We also documented clinical and biochemical data at the initiation of PD, including body mass index (BMI), blood pressure (BP), hemoglobin, serum ALB, CRP, ferritin, creatinine, blood urea nitrogen, total cholesterol (CHOL), high-density lipoprotein cholesterol (HDL-C), low-density lipoprotein cholesterol (LDL-C), triglyceride (TG), and lipoprotein(a) (Lp(a)) levels. We also collected KT/V data information, Which is combined. CAR was calculated as CRP (mg/L) divided by ALB (g/dL). The quantification of serum ALB was performed using the bromocresol green method, and serum CRP was determined by rate turbidity turbidimetry. The parameters used for quality control of all data were based on the patient’s standard serum, as outlined by the reference method for health industry standards in the People’s Republic of China. All baseline data were obtained during the first 1 to 3 months of PD. Baseline residual renal function was assessed using the estimated glomerular filtration rate (eGFR) as determined by the creatinine equation provided by the Chronic Kidney Disease Epidemiology Collaboration. Cardiovascular events were defined as the occurrence of first myocardial infarction, stroke, heart failure, unstable angina during hospitalization; peripheral vascular disease; sudden death; death related to cardiovascular surgery, ruptured aneurysm, or other CVD; fatal pulmonary embolism; and other cardiovascular or unknown causes of death [12]. This definition was established by a PD follow-up panel consisting of primary care nurses and professors with expertise in PD.

### Statistical analyses

Patients were classified into tertiles (Ts) based on their measured CAR levels: T1, ≤ 0.082 mg/g; T2, 0.082–0.227 mg/g; and T3, > 0.227 mg/g. Participant characteristics are listed according to CAR group. Results were expressed as frequencies and percentages for categorical variables, means and standard deviations (SDs) for normally distributed continuous variables, or medians and interquartile ranges for continuous variables that were not normally distributed. Chi-square, one-way analysis of variance (ANOVA), or Mann-Whitney U-tests were used to test for differences in categorical or continuous variables between patients from different CAR groups. The duration of survival was described using Kaplan–Meier curves, and differences in survival probabilities were compared between groups using the log-rank test. The association between CAR and mortality was examined using Cox proportional hazards models. We collected data including the timing of the switch to HD, receipt of a kidney transplant, transfer of care to another center, refusal of further treatment, and loss to follow-up. We first checked unadjusted associations between variables and then performed regression analyses, adjusting for age, sex, DM, CVD, BMI, hemoglobin, HDL-C, Kt/V, serum magnesium, total calcium, and ferritin. In order to examine whether CAR was independently associated with mortality, we added platelet (PLT) levels and neutrophil to lymphocyte ratio (N/L) to the regression models. A univariate Cox regression model was used to calculate the risk ratio (RR) for each indicator. Multivariate Cox regression models were used to analyze variables with statistically significant differences (*p* < .05) in univariate analyses and those considered to be related to mortality among patients with PD. Hazard ratio (HR) and 95% confidence interval (CI) were used to describe the strength and association of each variable with study outcome. All analyses were conducted using SPSS version 22.0 (SPSS, Inc., Chicago, IL). A *p* value < .05 was considered statistically significant.

## Results

**Figure 1.**
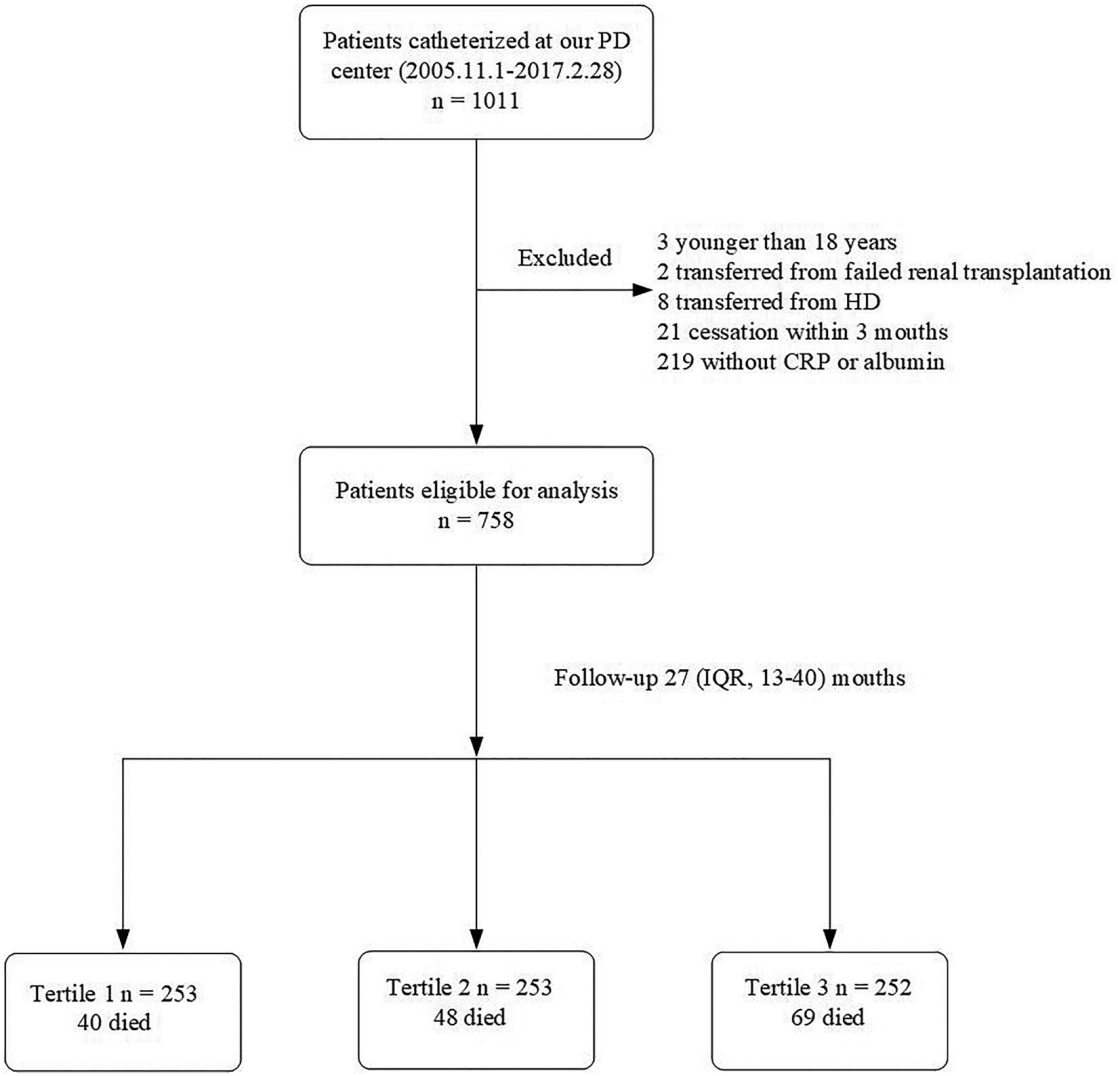
Flow-chart for the study. PD: peritoneal dialysis; HD: hemodialysis; CRP: C-reactive protein; IQR: interquartile range.

A total of 1011 incident patients with PD from our hospital were enrolled in the study. In total, 34 subjects were excluded: 3 subjects were < 18 years of age, 2 with failed renal allograft, 8 who were transferred from HD, and 21 who had received PD for less than 3 months. The medical records for another 219 patients did not include baseline measurements of CRP or ALB; these patients were also excluded. Ultimately, 758 patients qualified for inclusion in the study (Figure 1). The institutional protocol for PD dictates the use of 1.5% or 2.5% dextrose as PD dialysate and use of the twin-bag system for all patients with PD. Mean participant age was 49.1 ± 14.2 years. Among the study population, 56% were male, and 18.6% had DM (Table 1). Chronic glomerulonephritis was the most common cause of ESRD (64.8%), followed by diabetic nephropathy (15.6%) and hypertension (12.0%).

**Table 1.** Baseline characteristics of individuals stratified by CAR tertiles.

Variables	CAR			Total	*p*
	≤ 0.082 (*n* = 253)	0.082–0.227 (*n* = 253)	> 0.227 (*n* = 252)		
Age (year)	47.0 ± 13.1	48.1 ± 13.8	52.2 ± 15.1	49.1 ± 14.2	<.001
Men (%)	117 (46.2)	153 (60.5)	154 (61.1)	495 (56.0)	.001
Body mass index (kg/m^2^)	21.6 ± 3.2	22.4 ± 3.5	21.9 ± 3.4	22.0 ± 3.4	.039
Diabetes (%)	40 (15.8)	50 (19.8)	51 (20.2)	141 (18.6)	.373
CVD (%)	18 (7.1)	23 (9.1)	40 (15.9)	81 (10.7)	.004
Systolic blood pressure (mmHg)	144 ± 23	147 ± 25	147 ± 29	146 ± 26	.213
Diastolic blood pressure (mmHg)	88 ± 14	89 ± 15	88 ± 16	88 ± 16	.716
Total Kt/V	2.3 (1.8, 2.8)	2.2 (1.7, 2.7)	2.1 (1.7, 2.7)	2.2 (1.7, 2.7)	.298
eGFR (ml/min per 1.73 m^2^)	3.2 (1.9, 5.5)	3.3 (1.8, 5.7)	3.2 (1.3, 5.6)	3.27(1.67, 5.58)	.552
Hemoglobin (g/dL)	80.1 ± 15.5	80.5 ± 18.1	76.7 ± 14.9	79.07 ± 16.27	.016
White blood cell (×10^9^ g/L)	5.58 ± 1.78	6.1 ± 2.10	6.53 ± 1.47	6.08 ± 2.17	<.001
Platelet (×10^9^ g/L)	161 ± 69	168 ± 68	184 ± 86	170.99 ± 75.51	.002
N/L	3.17 (2.35, 4.60)	3.68 (2.71, 4.89)	3.89 (2.61, 5.77)	3.56 (2.52, 5.09)	<.001
P/L	140 (101, 175)	143 (103, 193)	155 (106, 215)	144 (103,197)	.006
Albumin (g/L)	37.2 ± 4.6	35.3 ± 5.2	33.6 ± 4.9	35.36 ± 5.10	<.001
C-reactive protein (mg/L)	1.80 (1.27, 2.38)	4.50 (3.61, 5.65)	20.90 (11.35, 44.18)	4.49 (2.32, 11.40)	<.001
Total cholesterol (mmol/L)	4.21 ± 1.10	4.19 ± 1.07	4.10 ± 1.14	4.16 ± 1.14	.506
HDL (mmol/L)	1.14 (0.94, 1.39)	1.09 (0.91, 1.44)	1.01 (0.83, 1.29)	1.08 (0.89, 1.36)	<.001
Lp(a) (mg/L )	277 (150, 486)	320 (162, 558)	422 (208, 697)	339 (170, 587)	<.001
Calcium (mmol/L)	2.00 ± 0.25	1.97 ± 0.27	1.94 ± 0.26	1.97 ± 0.26	.031
Magnesium (mmol/L)	1.01 ± 0.21	0.96 ± 0.19	0.92 ± 0.19	0.93(0.83, 1.07)	<.001
CAR (×10^–3^)	0.05 (0.03, 0.07)	0.13 (0.10, 0.17)	0.62 (0.34, 1.32)	0.13 (0.07, 0.34)	<.001
Ferritin (µg/L)	152 (76, 302)	223 (99, 429)	290 (136, 532)	214 (101, 430)	<.001

CAR, C-reactive protein to albumin ratio; CVD, cardiovascular disease; N/L, neutrophil-to-lymphocyte ratio; P/L, platelet-to-lymphocyte ratio; HDL, high-density lipoprotein; Lp(a), Lipoprotein(a). *p* < .05 was considered statistically significant.

### CAR predicted all-cause mortality during follow-up

Baseline CAR ranged from 0.007 to 9.967 (interquartile range [IQR] 0.07–0.34 mg/g, mean 0.42 mg/g). The clinical characteristics of participants, grouped according to CAR tertiles, are displayed in Table 1. Higher CAR levels were associated with male gender, and CVD, as well as increased age, BMI, PLT, N/L, PLT to lymphocyte ratio (P/L), CRP, and ferritin. Higher CAR levels were associated with decreases in levels of hemoglobin, ALB, HDL, calcium, and magnesium (*p* < .05). No significant differences were noted among patients of different CAR tertiles with regard to the prevalence of DM, total Kt/V, systolic BP, diastolic BP, TG, or eGFR (Table 1).

### Correlations between CAR and inflammation

As shown in Table 2, CAR was correlated with CRP (*r* = 0.99), with N/L and P/L values, and with PLT, white blood cell (WBC) and neutrophil counts (*r* = 0.098, 0.094, 0.128, 0.139, and 0.129, respectively). However, CAR was negatively correlated with serum ALB (r = −0.223; Table 2).

**Table 2.** Correlations between CAR and various parameters of inflammation.

	CAR	N/L	P/L	CRP	ALB	PLT	N
N/L	0.098^a^						
P/L	0.094^a^	0.587^a^					
CRP	0.987^a^	0.087^b^	0.074^b^				
ALB	–0.223^a^	–0.116^b^	–0.130^a^	–0.156^a^			
PLT	0.128^a^	0.044	0.640^a^	0.127^a^	–0.068		
N	0.129^a^	0676^a^	0.194^a^	0.184^a^	–0.094^b^	0.342^a^	
WBC	0.139^a^	0.484^a^	0.104^a^	0.131^a^	–0.092^b^	0.418^a^	0.945a

CAR: C-reactive protein-to-albumin ratio; N/L: neutrophil-to-lymphocyte ratio; P/L: platelet-to-lymphocyte ratio; CRP, C-reactive protein; ALB, albumin; PTL: platelets; N: neutrophils.

^a^Correlation is significant at the 0.01 level (2-tailed).

^b^Correlation is significant at the 0.05 level (2-tailed).

### Correlations between CAR and all-cause mortality

**Figure 2.**
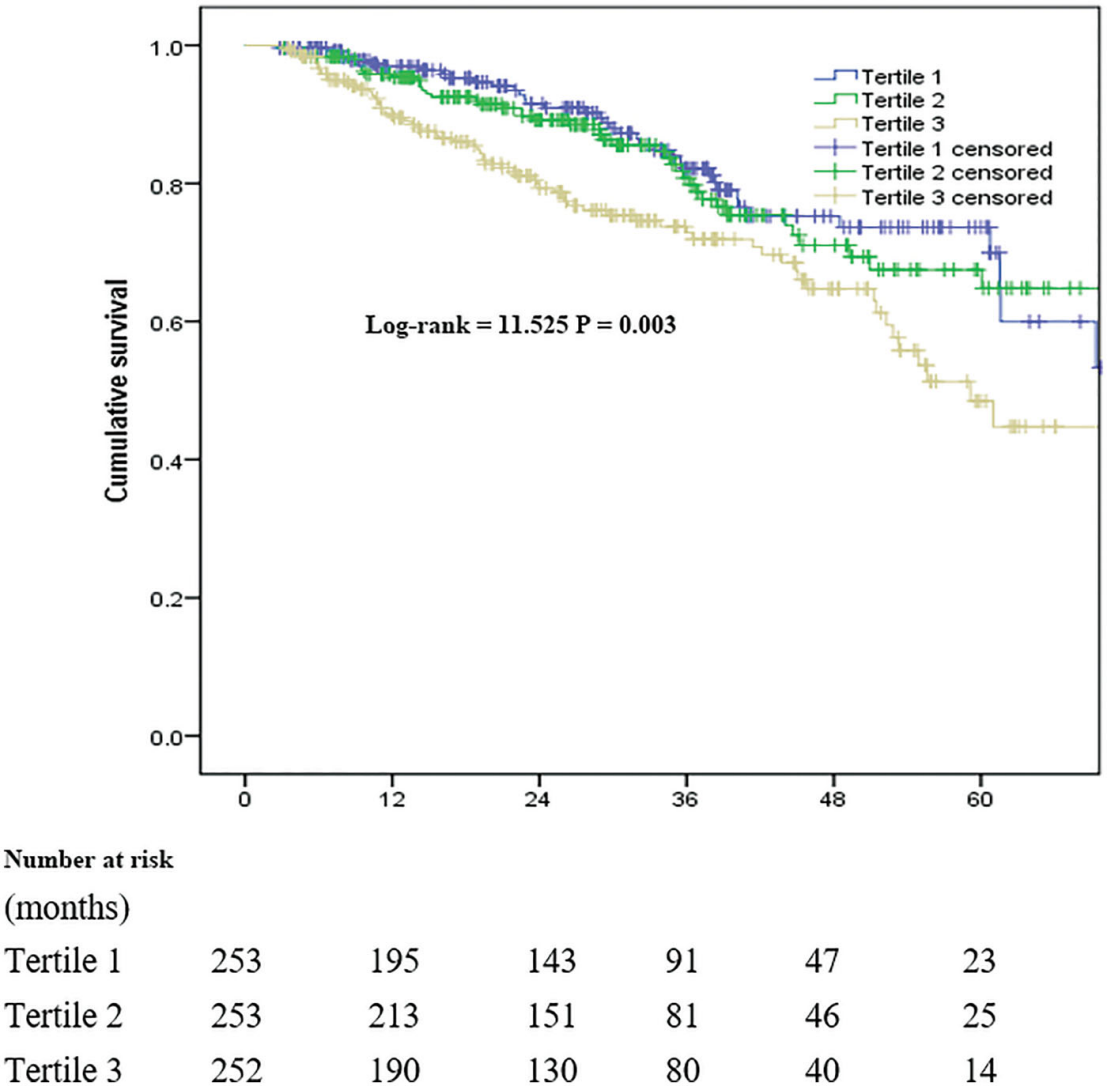
Survival curves for patient groups based on C-reactive protein to albumin ratio.

The median follow-up period was 27 months (IQR, 14–40 months), and 157 patients (20.7%) had died by the end of the follow-up period. Forty-three patients (5.7%) had undergone kidney transplantation, 106 (14.0%) were transferred to HD, 4 (0.5%) were transferred to other PD centers, and 15 (2.0%) discontinued follow-up; the remaining 433 patients (57.1%) continued to receive follow-up at our PD center. Among 157 cases of mortality, 87 were related to CVD (55.4%). The correlation between CAR and all-cause mortality is shown in Figure 2. Survival in the T1 group at the end of 1, 3, and 5 years was 96.9%, 82.2%, and 73.6%, respectively. Survival in the T2 group at the end of 1, 3, and 5 years was 95.4%, 80.8%, and 67.5%, respectively. Survival in the T3 group at the end of 1, 3, and 5 years was 90.0%, 79.3%, and 48.5%, respectively. The rate of survival among patients in the T3 group was significantly lower than that among patients in the T1 and T2 groups (*p* = 0.003). After adjustment for multiple variables, the results of Cox regression analysis showed that higher CAR was associated with increased all-cause mortality (Table 3). In model 3, for comparisons of patients from tertile 3 with those from tertile 1, the HR for all-cause mortality was 1.91 (95% CI: 1.05–3.47). The association between CAR and all-cause mortality remained significant when CAR was examined as a continuous variable. Furthermore, higher serum ALB correlated with increased all-cause mortality in models 1, 2, and 3. Higher serum CRP was correlated with increased all-cause mortality in model 1 but not in models 2 and 3 (Table 3). However, after adjust both CRP and ALB in CAR tertiles and continuous variable, it was found that there was no statistical difference (Table 4).

**Table 3.** Relationship between all-cause mortality and CRP, ALB, and CAR (Tertile 3 vs. Tertile1).

Variables	CRP	*p*-value	ALB	*p*-value	CAR^d^	*p*-value	Continuous CAR	*p*-value
	HR (95% CI)		HR (95% CI)	P-value	HR (95% CI)		HR (95% CI)	
All-cause mortality								
Model 1^a^	1.01 (1.00–1.01)	<.001	0.95 (0.92–0.98)	<0.001	1.83 (1.24–2.70)	0.003	1.32 (1.16–1.49)	<0.001
Model 2^b^	1.01 (1.00–1.01)	.124	0.94 (0.90–0.98)	0.002	1.93 (1.08–3.46)	0.027	1.24 (1.05–1.48)	0.014
Model 3^c^	1.01 (1.00–1.01)	.074	0.93 (0.89–0.97)	0.002	1.91 (1.05–3.47)	0.034	1.21 (1.00–1.46)	0.046

CAR: C-reactive protein to albumin ratio; CRP: C-reactive protein; ALB: albumin; BMI, body mass index.

^a^Model 1: Unadjusted.

^b^Model 2: Adjusted for age, sex, diabetes, cardiovascular diseases, BMI, hemoglobin, Kt/V, high-density lipoprotein, magnesium, total calcium, and ferritin.

^c^Model 3: Model 2 plus adjustment for platelets and neutrophil-to-lymphocyte ratio.

^d^Tertile 3 vs. tertile 1.

**Table 4.** Relationship between all-cause mortality and CAR after adjust CRP and ALB (Tertile 3 vs. Tertile1).

Variables	CAR^d^	*p*-value	Continuous CAR	*p*-value
	HR (95% CI)		HR (95% CI)	
All-cause mortality				
Model 1^a^	1.28 (0.82–2.02)	.280	1.72 (0.61–4.85)	.302
Model 2^b^	1.47 (0.76–2.83)	.252	0.90 (0.20–4.14)	.893
Model 3^c^	1.39(0.71–2.73)	.335	1.16(0.25–5.34)	.849

CAR: C-reactive protein to albumin ratio; CRP: C-reactive protein; ALB: albumin; BMI, body mass index.

^a^Model 1: Adjusted for CRP and ALB.

^b^Model 2: Adjusted for age, sex, diabetes, cardiovascular diseases, BMI, hemoglobin, Kt/V, high-density lipoprotein, magnesium, total calcium, and ferritin.

^c^Model 3: Model 2 plus adjustment for platelets and neutrophil-to-lymphocyte ratio.

^d^Tertile 3 vs. tertile 1.

## Discussion

In this study, we examined the correlation between CAR and clinical characteristics of patients with PD. We found that higher CAR was strongly associated with increased all-cause mortality. To the best of our knowledge, this finding has not previously been reported.

Patients with chronic kidney disease (CKD) tend to exhibit persistent inflammation [13]. CRP is an acute-phase reactant produced primarily by the liver in the presence of acute and chronic inflammation. Prior studies suggested that CRP was an important risk factor for increased cardiovascular mortality among patients under HD or PD [14]. Chen et al. found that, among patients with PD, CRP was an independent predictor for higher all-cause mortality and increased risk of developing major adverse cardiovascular events. Among HD patients, higher serum CRP levels were associated with an increased risk of all-cause mortality but not with major adverse cardiovascular events [15]. On the contrary, we did not find any correlation between serum CRP and any of the adverse events listed above. Plausible reasons for the lack of such an association may include differences between data-sets in clinical characteristics or the duration of follow-up. This should be clarified in future studies.

On the other hand, ALB serves as an indicator of an individual’s nutritional status. ALB is the most abundant serum protein, accounting for 70% of osmotic pressure. ALB is also the most extensively studied serum protein in studies of maintenance dialysis patients [16]. However, the relationship between ALB and mortality in these patients remains controversial. In patients undergoing PD, initial serum ALB levels are closely related to cardiovascular [17] and all-cause mortality [18–20]. However, a study carried out by Malgorzewicz et al. [21] reported different findings after adjusting for confounders. In both models employed for this study, hypoalbuminemia was shown to be not only a risk factor for higher all-cause mortality but also for all-cause mortality among patients with PD. In patients with PD, hypoproteinemia can be multifactorial and may indicate the presence of increased systemic inflammation, volume overload, ongoing peritoneal and urinary protein loss, or a compensatory response to impaired hepatic ALB synthesis [22].

When analyzed in combination, CRP and ALB not only provide information on both inflammation and nutrition but may also be used to predict patient prognosis [23,24]. Previous studies have shown that CAR is more effective for predicting prognosis than CRP or ALB alone [25]. As a prognostic marker, CAR has been extensively studied in patients with active infections, malignancies, and other diseases [5,10]. In one study involving elderly patients hospitalized in the emergency department, the ratio of highly sensitive CRP to albumin at admission was associated with increased all-cause mortality in patients > 65 years of age [23]. CAR has been found to be a predictor of increased mortality in patients with acute pancreatitis [26] or various malignancies [10,27,28]. Consistent with these findings, the results presented above show that all-cause mortality was higher in PD patients stratified to higher CAR tertiles. According to protocols in place at our research center, the reference range for CAR is 0–0.2 (×10^−3^). We have also shown that all-cause mortality was higher in tertile 3 than in tertile 1 and tertile 2. We extended the utility of these findings by combining CRP with ALB to improve the accuracy of prognosis predictions in the treatment of patients with PD. The advantages of using CAR instead of CRP or ALB alone include the following: first, levels of inflammatory markers such as CRP and ALB may vary significantly among individuals, depending on the severity of inflammation. In addition, the ratio of CRP to ALB may be used to concurrently assess inflammation and nutrition. Use of this measure, compared to CRP or ALB alone, is expected to improve the accuracy of prognostic predictions.

Our study had some limitations. First, this was a single-center retrospective study, and the existence of center-specific effects cannot be completely excluded. Second, we collected baseline CAR only and did not consider the effects of temporal changes in CAR during follow-up. Third, we did not exclude those who were received infusions of human albumin to treat malnutrition. Finally, our sample size was limited, and un-recognized confounding factors may have gone unnoticed. Therefore, the possibility of residual confounding could not be eliminated. Future research should address these issues.

In conclusion, CAR is a simple, inexpensive biomarker that yields reproducible results. CAR may be used as an effective outcome predictor among patients with PD. CAR is an independent risk factor for all-cause mortality in these patients.

## Data Availability

All data generated or analyzed during this study have been included in this manuscript.
